# Isoprop­yl (3,4-dimethyl-5,5-dioxo-4*H*-pyrazolo­[4,3-*c*][1,2]benzothia­zin-2-yl)acetate

**DOI:** 10.1107/S1600536810037797

**Published:** 2010-09-25

**Authors:** Sana Aslam, Matloob Ahmad, Hamid Latif Siddiqui, Masood Parvez

**Affiliations:** a Institute of Chemistry, University of the Punjab, Lahore 54590, Pakistan; b Applied Chemistry Research Centre, PCSIR Laboratories Complex, Lahore 54600, Pakistan; c Department of Chemistry, The University of Calgary, 2500 University Drive NW, Calgary, Alberta, Canada T2N 1N4

## Abstract

In the title mol­ecule, C_16_H_19_N_3_O_4_S, the heterocyclic thia­zine ring adopts a half-chair conformation, with the S and N atoms displaced by 0.547 (2) and −0.254 (3) Å, respectively, from the plane formed by the remaining atoms. In the crystal, weak C—H⋯N and C—H⋯O hydrogen bonds link the mol­ecules.

## Related literature

For the biological applications of benzothia­zines, see: Shavel *et al.* (1968 ▶); Krapcho (1969 ▶); Lombardino & Wiseman (1972 ▶); Kwon & Park (1996 ▶); Wells *et al.* (2001 ▶); Zia-ur-Rehman *et al.* (2006 ▶); Ahmad *et al.* (2010 ▶). For related structures, see: Siddiqui *et al.* (2008 ▶). For ring puckering parameters, see: Cremer & Pople (1975 ▶).
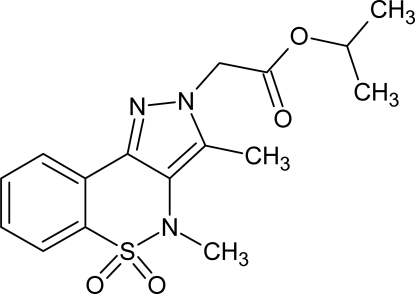


## Experimental

### 

#### Crystal data

C_16_H_19_N_3_O_4_S
*M* *_r_* = 349.40
Triclinic, 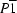

*a* = 7.6370 (2) Å
*b* = 8.5412 (2) Å
*c* = 13.3096 (4) Å
α = 98.4584 (18)°
β = 97.4870 (13)°
γ = 95.9599 (17)°
*V* = 844.69 (4) Å^3^
*Z* = 2
Mo *K*α radiation
μ = 0.22 mm^−1^
*T* = 173 K
0.18 × 0.16 × 0.11 mm

#### Data collection

Nonius Kappa CCD diffractometer
Absorption correction: multi-scan (*SORTAV*; Blessing, 1997 ▶) *T*_min_ = 0.962, *T*_max_ = 0.977
12392 measured reflections
3824 independent reflections
3552 reflections with *I* > 2.0σ(*I*)
*R*_int_ = 0.017

#### Refinement

*R*[*F* ^2^ > 2σ(*F* ^2^)] = 0.037
*wR*(*F* ^2^) = 0.095
*S* = 1.04
3824 reflections
221 parameters
H-atom parameters constrained
Δρ_max_ = 0.38 e Å^−3^
Δρ_min_ = −0.38 e Å^−3^

### 

Data collection: *COLLECT* (Hooft, 1998 ▶); cell refinement: *HKL DENZO* (Otwinowski & Minor, 1997 ▶); data reduction: *SCALEPACK* (Otwinowski & Minor, 1997 ▶); program(s) used to solve structure: *SHELXS97* (Sheldrick, 2008 ▶); program(s) used to refine structure: *SHELXL97* (Sheldrick, 2008 ▶); molecular graphics: *ORTEP-3 for Windows* (Farrugia, 1997 ▶); software used to prepare material for publication: *SHELXL97*.

## Supplementary Material

Crystal structure: contains datablocks global, I. DOI: 10.1107/S1600536810037797/hb5643sup1.cif

Structure factors: contains datablocks I. DOI: 10.1107/S1600536810037797/hb5643Isup2.hkl

Additional supplementary materials:  crystallographic information; 3D view; checkCIF report

## Figures and Tables

**Table 1:** Hydrogen-bond geometry (Å, °)

*D*—H⋯*A*	*D*—H	H⋯*A*	*D*⋯*A*	*D*—H⋯*A*
C4—H4⋯O2^i^	0.95	2.54	3.4015 (17)	150
C12—H12*A*⋯N2^ii^	0.99	2.50	3.4413 (17)	158
C12—H12*B*⋯O1^iii^	0.99	2.51	3.4694 (17)	163
C15—H15*B*⋯O3^iv^	0.98	2.53	3.456 (2)	157

Symmetry codes: (i) 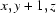; (ii) 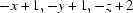; (iii) 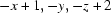; (iv) 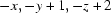.

**Fig. 1.**
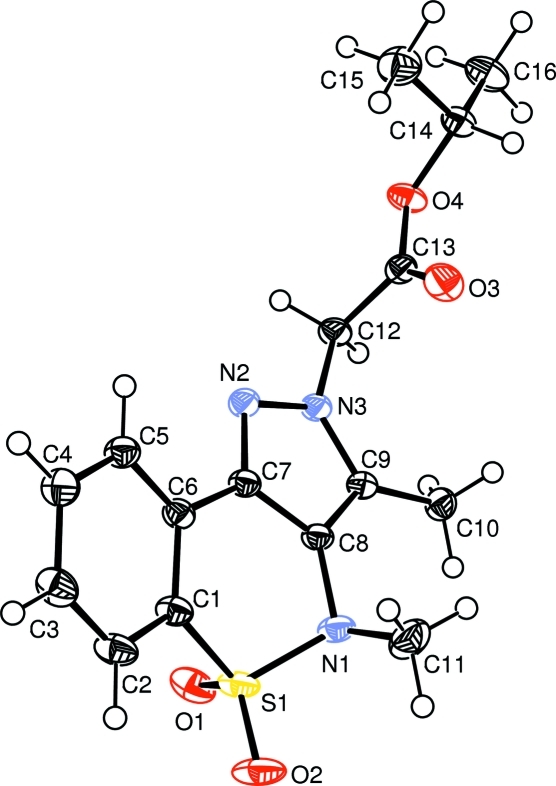
The title molecule with the displacement ellipsoids at the 50% probability level.

## supplementary crystallographic information

### Comment

Important anti-inflammatory and analgesic properties of 4-hydroxy-2*H*-1,2-benzothiazine-3-carboxamide 1,1-dioxides (Oxicams) (Lombardino & Wiseman, 1972; Kwon & Park, 1996) boosted research interests in benzothiazines. These studies led to discovery of a wide range of benzothiazine derivatives having potential biological activities, such as inhibitors of calpain I belonging to a family of calcium-dependent, non-lysosomal cysteine proteases (proteolytic enzymes) (Wells *et al.*, 2001), antifungal (Shavel *et al.*, 1968) and antibacterial agents (Zia-ur-Rehman *et al.*, 2006), central nervous system depressants (drugs used to slow down brain activity and are used to treat anxiety, muscle tension, pain, insomnia, acute stress reactions, panic attacks and seizure disorders), tranquilizers (Krapcho, 1969) as well as antioxidants (Ahmad *et al.*, 2010).

In the title molecule (Fig. 1), the heterocyclic thiazine ring adopts a half-chair conformation, with atoms S1 and N1 displaced from the plane C1\C6\C7\C8 by 0.547 (2) and -0.254 (3) Å, respectively. The pertinent puckering parameters (Cremer & Pople, 1975) are: *Q* = 0.5288 (10) Å, θ = 63.93 (13)° and φ = 19.41 (16)°. Similar conformations of the corresponding rings have been reported in some closely related molecules (Siddiqui *et al.*, 2008). The intermolecular interactions of the type C—H···N and C—H···O are listed in Tab. 1.

### Experimental

A mixture of 3,4-dimethyl-2,4-dihydropyrazolo[4,3-*c*][1,2]benzothiazine 5,5-dioxide (5.00 g, 0.020 mol), anhydrous potassium carbonate (3.31 g, 0.024 mol), isopropyl chloroacetate (3.28 g, 0.024 mol) and acetonitrile (30 ml) was refluxed for 10 h at 355 K, *i. e.* at the boiling point of acetonitrile. The completion of reaction was monitored by thin layer chromatography (TLC). After completion of the reaction the solvent was removed under vacuum. The residue was washed with cold water to obtain the title compound as a white crystalline product. Yellow prisms of (I) of approximate size 0.10–0.30 mm were grown from a solution of 0.5 g of the title compound dissolved in 15 ml mixture of methanol and chloroform (1:1); the methanol contained 0.5% of moisture. Yield of the recrystallised product : 80%

### Refinement

All the hydrogen atoms were discernible in the difference electron density map. However, they were positioned into the idealized positions and refined by the riding-model approximation. Used constraints: C—H = 0.98, 0.99, 1.00 and 0.95 Å for methyl, methylene, methine and aryl H-atoms, respectively. *U*_iso_(H) = 1.5*U*_eq_(methyl C-atoms) and 1.2*U*_eq_(non-methyl C-atoms). The methyl groups were allowed to rotate about their axes during the refinement.

### Crystal data

**Table:**

C_16_H_19_N_3_O_4_S	*Z* = 2
*M**_r_* = 349.40	*F*(000) = 368
Triclinic, *P*1	*D*_x_ = 1.374 Mg m^−^^3^
Hall symbol: -P 1	Melting point = 453–455 K
*a* = 7.6370 (2) Å	Mo *K*α radiation, λ = 0.71073 Å
*b* = 8.5412 (2) Å	Cell parameters from 3658 reflections
*c* = 13.3096 (4) Å	θ = 1.0–27.5°
α = 98.4584 (18)°	µ = 0.22 mm^−^^1^
β = 97.4870 (13)°	*T* = 173 K
γ = 95.9599 (17)°	Prism, yellow
*V* = 844.69 (4) Å^3^	0.18 × 0.16 × 0.11 mm

### Data collection

**Table:**

Nonius Kappa CCD diffractometer	3824 independent reflections
Radiation source: fine-focus sealed tube	3552 reflections with *I* > 2.0σ(*I*)
graphite	*R*_int_ = 0.017
ω and φ scans	θ_max_ = 27.5°, θ_min_ = 2.7°
Absorption correction: multi-scan (*SORTAV*; Blessing, 1997)	*h* = −9→9
*T*_min_ = 0.962, *T*_max_ = 0.977	*k* = −11→11
12392 measured reflections	*l* = −17→17

### Refinement

**Table:**

Refinement on *F*^2^	Primary atom site location: structure-invariant direct methods
Least-squares matrix: full	Secondary atom site location: difference Fourier map
*R*[*F*^2^ > 2σ(*F*^2^)] = 0.037	Hydrogen site location: difference Fourier map
*wR*(*F*^2^) = 0.095	H-atom parameters constrained
*S* = 1.04	*w* = 1/[σ^2^(*F*_o_^2^) + (0.0415*P*)^2^ + 0.4374*P*] where *P* = (*F*_o_^2^ + 2*F*_c_^2^)/3
3824 reflections	(Δ/σ)_max_ = 0.001
221 parameters	Δρ_max_ = 0.38 e Å^−^^3^
0 restraints	Δρ_min_ = −0.38 e Å^−^^3^
72 constraints	

### Special details

**Table:**

Experimental. Analysis of the title compound by EI (electron impact) and MS (mass spectrometry): EI—MS (*m/z*, *i. e.* mass to charge ratio): 349.1
Geometry. All esds (except the esd in the dihedral angle between two l.s. planes) are estimated using the full covariance matrix. The cell esds are taken into account individually in the estimation of esds in distances, angles and torsion angles; correlations between esds in cell parameters are only used when they are defined by crystal symmetry. An approximate (isotropic) treatment of cell esds is used for estimating esds involving l.s. planes.
Refinement. Refinement of *F*^2^ against ALL reflections. The weighted *R*-factor *wR* and goodness of fit *S* are based on *F*^2^, conventional *R*-factors *R* are based on *F*, with *F* set to zero for negative *F*^2^. The threshold expression of *F*^2^ > σ(*F*^2^) is used only for calculating *R*-factors(gt) *etc*. and is not relevant to the choice of reflections for refinement. *R*-factors based on *F*^2^ are statistically about twice as large as those based on *F*, and *R*- factors based on ALL data will be even larger.

### Fractional atomic coordinates and isotropic or equivalent isotropic displacement parameters (Å^2^)

**Table:**

	*x*	*y*	*z*	*U*_iso_*/*U*_eq_	
S1	0.35224 (5)	−0.09565 (4)	0.67475 (2)	0.02666 (11)	
O1	0.53320 (15)	−0.09803 (13)	0.71900 (8)	0.0344 (3)	
O2	0.26989 (18)	−0.21974 (12)	0.59324 (8)	0.0400 (3)	
O3	0.07421 (14)	0.34287 (14)	1.06534 (8)	0.0343 (2)	
O4	0.25929 (13)	0.47365 (12)	1.20503 (7)	0.0266 (2)	
N1	0.23204 (16)	−0.09429 (13)	0.76983 (8)	0.0248 (2)	
N2	0.38590 (15)	0.30879 (13)	0.90139 (8)	0.0220 (2)	
N3	0.35630 (15)	0.22831 (13)	0.97981 (8)	0.0211 (2)	
C1	0.33526 (18)	0.09155 (15)	0.63524 (10)	0.0228 (3)	
C2	0.3215 (2)	0.10488 (17)	0.53157 (10)	0.0280 (3)	
H2	0.3125	0.0127	0.4808	0.034*	
C3	0.3213 (2)	0.25514 (18)	0.50375 (11)	0.0290 (3)	
H3	0.3141	0.2664	0.4334	0.035*	
C4	0.33137 (19)	0.38921 (17)	0.57839 (10)	0.0266 (3)	
H4	0.3320	0.4917	0.5587	0.032*	
C5	0.34052 (18)	0.37494 (15)	0.68136 (10)	0.0234 (3)	
H5	0.3450	0.4674	0.7315	0.028*	
C6	0.34316 (17)	0.22555 (15)	0.71176 (9)	0.0204 (2)	
C7	0.34179 (17)	0.19804 (15)	0.81758 (9)	0.0200 (2)	
C8	0.28614 (17)	0.04849 (14)	0.84320 (9)	0.0199 (2)	
C9	0.29516 (17)	0.07097 (15)	0.94854 (9)	0.0202 (2)	
C10	0.25095 (18)	−0.04193 (16)	1.01953 (10)	0.0244 (3)	
H10A	0.2407	−0.1518	0.9834	0.037*	
H10B	0.1377	−0.0222	1.0431	0.037*	
H10C	0.3454	−0.0260	1.0788	0.037*	
C11	0.0369 (2)	−0.13282 (19)	0.73806 (13)	0.0377 (4)	
H11A	−0.0216	−0.1382	0.7990	0.057*	
H11B	0.0111	−0.2360	0.6923	0.057*	
H11C	−0.0077	−0.0497	0.7019	0.057*	
C12	0.38752 (18)	0.31656 (15)	1.08332 (9)	0.0226 (3)	
H12A	0.4809	0.4079	1.0880	0.027*	
H12B	0.4313	0.2466	1.1316	0.027*	
C13	0.21988 (18)	0.37767 (15)	1.11442 (9)	0.0218 (3)	
C14	0.11863 (18)	0.56384 (16)	1.24023 (10)	0.0244 (3)	
H14	0.0006	0.4963	1.2204	0.029*	
C15	0.1178 (2)	0.71022 (19)	1.18913 (13)	0.0372 (3)	
H15A	0.0255	0.7727	1.2119	0.056*	
H15B	0.0933	0.6778	1.1144	0.056*	
H15C	0.2343	0.7753	1.2079	0.056*	
C16	0.1623 (2)	0.6019 (2)	1.35555 (11)	0.0361 (3)	
H16A	0.0724	0.6629	1.3830	0.054*	
H16B	0.2798	0.6651	1.3747	0.054*	
H16C	0.1632	0.5024	1.3840	0.054*	

### Atomic displacement parameters (Å^2^)

**Table:**

	*U*^11^	*U*^22^	*U*^33^	*U*^12^	*U*^13^	*U*^23^
S1	0.0442 (2)	0.01991 (17)	0.01619 (16)	0.01115 (14)	0.00383 (13)	−0.00046 (12)
O1	0.0450 (6)	0.0374 (6)	0.0255 (5)	0.0218 (5)	0.0079 (4)	0.0062 (4)
O2	0.0736 (8)	0.0228 (5)	0.0202 (5)	0.0096 (5)	0.0019 (5)	−0.0054 (4)
O3	0.0281 (5)	0.0419 (6)	0.0263 (5)	−0.0015 (4)	0.0008 (4)	−0.0087 (4)
O4	0.0284 (5)	0.0302 (5)	0.0182 (4)	0.0082 (4)	−0.0001 (4)	−0.0060 (4)
N1	0.0368 (6)	0.0175 (5)	0.0177 (5)	0.0010 (4)	0.0021 (4)	−0.0018 (4)
N2	0.0290 (6)	0.0198 (5)	0.0163 (5)	0.0003 (4)	0.0044 (4)	0.0011 (4)
N3	0.0277 (5)	0.0197 (5)	0.0145 (5)	0.0004 (4)	0.0042 (4)	−0.0006 (4)
C1	0.0296 (7)	0.0217 (6)	0.0172 (6)	0.0072 (5)	0.0028 (5)	0.0015 (5)
C2	0.0383 (8)	0.0281 (7)	0.0171 (6)	0.0100 (6)	0.0028 (5)	−0.0005 (5)
C3	0.0368 (8)	0.0342 (7)	0.0178 (6)	0.0101 (6)	0.0037 (5)	0.0063 (5)
C4	0.0311 (7)	0.0264 (7)	0.0233 (6)	0.0056 (5)	0.0027 (5)	0.0071 (5)
C5	0.0272 (6)	0.0210 (6)	0.0209 (6)	0.0025 (5)	0.0027 (5)	0.0009 (5)
C6	0.0216 (6)	0.0222 (6)	0.0166 (6)	0.0027 (5)	0.0024 (4)	0.0010 (5)
C7	0.0235 (6)	0.0186 (6)	0.0173 (6)	0.0032 (5)	0.0033 (5)	0.0003 (4)
C8	0.0245 (6)	0.0176 (6)	0.0168 (6)	0.0028 (5)	0.0034 (5)	−0.0004 (4)
C9	0.0221 (6)	0.0193 (6)	0.0183 (6)	0.0025 (4)	0.0030 (5)	0.0004 (4)
C10	0.0284 (7)	0.0242 (6)	0.0205 (6)	0.0006 (5)	0.0041 (5)	0.0050 (5)
C11	0.0412 (9)	0.0314 (8)	0.0333 (8)	−0.0118 (6)	−0.0001 (7)	−0.0021 (6)
C12	0.0280 (6)	0.0230 (6)	0.0147 (6)	0.0017 (5)	0.0021 (5)	−0.0017 (5)
C13	0.0294 (7)	0.0198 (6)	0.0148 (6)	−0.0004 (5)	0.0030 (5)	0.0014 (4)
C14	0.0255 (6)	0.0270 (6)	0.0201 (6)	0.0064 (5)	0.0040 (5)	−0.0007 (5)
C15	0.0422 (9)	0.0327 (8)	0.0389 (8)	0.0111 (6)	0.0045 (7)	0.0096 (6)
C16	0.0431 (9)	0.0434 (9)	0.0209 (7)	0.0126 (7)	0.0056 (6)	−0.0033 (6)

### Geometric parameters (Å, °)

**Table:**

S1—O2	1.4288 (11)	C6—C7	1.4628 (17)
S1—O1	1.4333 (12)	C7—C8	1.4077 (17)
S1—N1	1.6567 (12)	C8—C9	1.3784 (17)
S1—C1	1.7670 (13)	C9—C10	1.4901 (17)
O3—C13	1.1990 (17)	C10—H10A	0.9800
O4—C13	1.3343 (15)	C10—H10B	0.9800
O4—C14	1.4710 (16)	C10—H10C	0.9800
N1—C8	1.4323 (15)	C11—H11A	0.9800
N1—C11	1.484 (2)	C11—H11B	0.9800
N2—C7	1.3335 (16)	C11—H11C	0.9800
N2—N3	1.3613 (15)	C12—C13	1.5153 (19)
N3—C9	1.3608 (16)	C12—H12A	0.9900
N3—C12	1.4473 (15)	C12—H12B	0.9900
C1—C2	1.3924 (18)	C14—C16	1.5066 (19)
C1—C6	1.4062 (17)	C14—C15	1.509 (2)
C2—C3	1.387 (2)	C14—H14	1.0000
C2—H2	0.9500	C15—H15A	0.9800
C3—C4	1.390 (2)	C15—H15B	0.9800
C3—H3	0.9500	C15—H15C	0.9800
C4—C5	1.3870 (18)	C16—H16A	0.9800
C4—H4	0.9500	C16—H16B	0.9800
C5—C6	1.3955 (18)	C16—H16C	0.9800
C5—H5	0.9500		
			
O2—S1—O1	119.34 (7)	C8—C9—C10	131.29 (12)
O2—S1—N1	108.09 (7)	C9—C10—H10A	109.5
O1—S1—N1	106.69 (6)	C9—C10—H10B	109.5
O2—S1—C1	109.45 (6)	H10A—C10—H10B	109.5
O1—S1—C1	108.09 (7)	C9—C10—H10C	109.5
N1—S1—C1	104.11 (6)	H10A—C10—H10C	109.5
C13—O4—C14	117.22 (10)	H10B—C10—H10C	109.5
C8—N1—C11	114.81 (11)	N1—C11—H11A	109.5
C8—N1—S1	109.87 (9)	N1—C11—H11B	109.5
C11—N1—S1	115.46 (10)	H11A—C11—H11B	109.5
C7—N2—N3	103.99 (10)	N1—C11—H11C	109.5
C9—N3—N2	113.73 (10)	H11A—C11—H11C	109.5
C9—N3—C12	128.00 (11)	H11B—C11—H11C	109.5
N2—N3—C12	118.25 (10)	N3—C12—C13	111.71 (11)
C2—C1—C6	121.72 (12)	N3—C12—H12A	109.3
C2—C1—S1	120.34 (10)	C13—C12—H12A	109.3
C6—C1—S1	117.89 (10)	N3—C12—H12B	109.3
C3—C2—C1	118.87 (13)	C13—C12—H12B	109.3
C3—C2—H2	120.6	H12A—C12—H12B	107.9
C1—C2—H2	120.6	O3—C13—O4	125.56 (13)
C2—C3—C4	120.23 (13)	O3—C13—C12	124.87 (12)
C2—C3—H3	119.9	O4—C13—C12	109.56 (11)
C4—C3—H3	119.9	O4—C14—C16	106.21 (11)
C5—C4—C3	120.70 (13)	O4—C14—C15	108.03 (12)
C5—C4—H4	119.7	C16—C14—C15	113.37 (13)
C3—C4—H4	119.7	O4—C14—H14	109.7
C4—C5—C6	120.35 (12)	C16—C14—H14	109.7
C4—C5—H5	119.8	C15—C14—H14	109.7
C6—C5—H5	119.8	C14—C15—H15A	109.5
C5—C6—C1	118.10 (12)	C14—C15—H15B	109.5
C5—C6—C7	124.07 (11)	H15A—C15—H15B	109.5
C1—C6—C7	117.70 (11)	C14—C15—H15C	109.5
N2—C7—C8	111.10 (11)	H15A—C15—H15C	109.5
N2—C7—C6	125.65 (11)	H15B—C15—H15C	109.5
C8—C7—C6	123.21 (11)	C14—C16—H16A	109.5
C9—C8—C7	106.45 (11)	C14—C16—H16B	109.5
C9—C8—N1	129.33 (11)	H16A—C16—H16B	109.5
C7—C8—N1	124.21 (11)	C14—C16—H16C	109.5
N3—C9—C8	104.72 (11)	H16A—C16—H16C	109.5
N3—C9—C10	123.98 (11)	H16B—C16—H16C	109.5
			
O2—S1—N1—C8	−167.41 (9)	C1—C6—C7—N2	164.83 (13)
O1—S1—N1—C8	63.09 (10)	C5—C6—C7—C8	158.23 (13)
C1—S1—N1—C8	−51.10 (10)	C1—C6—C7—C8	−17.56 (19)
O2—S1—N1—C11	−35.64 (12)	N2—C7—C8—C9	0.78 (15)
O1—S1—N1—C11	−165.14 (10)	C6—C7—C8—C9	−177.14 (12)
C1—S1—N1—C11	80.68 (11)	N2—C7—C8—N1	−179.36 (12)
C7—N2—N3—C9	−0.01 (14)	C6—C7—C8—N1	2.7 (2)
C7—N2—N3—C12	−178.48 (11)	C11—N1—C8—C9	83.07 (17)
O2—S1—C1—C2	−26.58 (14)	S1—N1—C8—C9	−144.81 (12)
O1—S1—C1—C2	104.88 (12)	C11—N1—C8—C7	−96.76 (15)
N1—S1—C1—C2	−141.94 (12)	S1—N1—C8—C7	35.36 (16)
O2—S1—C1—C6	155.87 (11)	N2—N3—C9—C8	0.48 (15)
O1—S1—C1—C6	−72.67 (12)	C12—N3—C9—C8	178.77 (12)
N1—S1—C1—C6	40.51 (12)	N2—N3—C9—C10	−179.31 (12)
C6—C1—C2—C3	2.0 (2)	C12—N3—C9—C10	−1.0 (2)
S1—C1—C2—C3	−175.44 (11)	C7—C8—C9—N3	−0.72 (14)
C1—C2—C3—C4	−1.1 (2)	N1—C8—C9—N3	179.42 (13)
C2—C3—C4—C5	−0.5 (2)	C7—C8—C9—C10	179.05 (13)
C3—C4—C5—C6	1.2 (2)	N1—C8—C9—C10	−0.8 (2)
C4—C5—C6—C1	−0.4 (2)	C9—N3—C12—C13	−85.83 (16)
C4—C5—C6—C7	−176.15 (12)	N2—N3—C12—C13	92.39 (14)
C2—C1—C6—C5	−1.3 (2)	C14—O4—C13—O3	−10.09 (19)
S1—C1—C6—C5	176.25 (10)	C14—O4—C13—C12	170.83 (11)
C2—C1—C6—C7	174.78 (13)	N3—C12—C13—O3	8.23 (18)
S1—C1—C6—C7	−7.70 (17)	N3—C12—C13—O4	−172.68 (10)
N3—N2—C7—C8	−0.47 (14)	C13—O4—C14—C16	155.92 (12)
N3—N2—C7—C6	177.38 (12)	C13—O4—C14—C15	−82.14 (14)
C5—C6—C7—N2	−19.4 (2)		

### Hydrogen-bond geometry (Å, °)

**Table:**

*D*—H···*A*	*D*—H	H···*A*	*D*···*A*	*D*—H···*A*
C4—H4···O2^i^	0.95	2.54	3.4015 (17)	150
C12—H12A···N2^ii^	0.99	2.50	3.4413 (17)	158
C12—H12B···O1^iii^	0.99	2.51	3.4694 (17)	163
C15—H15B···O3^iv^	0.98	2.53	3.456 (2)	157

Symmetry codes: (i) *x*, *y*+1, *z*; (ii) −*x*+1, −*y*+1, −*z*+2; (iii) −*x*+1, −*y*, −*z*+2; (iv) −*x*, −*y*+1, −*z*+2.
